# Establishment and validation of a pseudovirus neutralization assay for SARS-CoV-2

**DOI:** 10.1080/22221751.2020.1743767

**Published:** 2020-03-24

**Authors:** Jianhui Nie, Qianqian Li, Jiajing Wu, Chenyan Zhao, Huan Hao, Huan Liu, Li Zhang, Lingling Nie, Haiyang Qin, Meng Wang, Qiong Lu, Xiaoyu Li, Qiyu Sun, Junkai Liu, Changfa Fan, Weijin Huang, Miao Xu, Youchun Wang

**Affiliations:** a Division of HIV/AIDS and Sex-transmitted Virus Vaccines, Institute for Biological Product Control, National Institutes for Food and Drug Control (NIFDC), Beijing, People’s Republic of China; b Graduate School of Peking Union Medical College, Beijing, People’s Republic of China; c Wuhan Institute of Biological Products, Wuhan, People’s Republic of China; d Division of Animal Model Research, Institute for Laboratory Animal Resources, National Institutes for Food and Drug Control, Beijing, People’s Republic of China; e Institute for Biological Product Control, National Institutes for Food and Drug Control, Beijing, People’s Republic of China

**Keywords:** SARS-CoV-2, COVID-19, neutralizing antibody, pseudovirus, neutralization assay

## Abstract

Pseudoviruses are useful virological tools because of their safety and versatility, especially for emerging and re-emerging viruses. Due to its high pathogenicity and infectivity and the lack of effective vaccines and therapeutics, live SARS-CoV-2 has to be handled under biosafety level 3 conditions, which has hindered the development of vaccines and therapeutics. Based on a VSV pseudovirus production system, a pseudovirus-based neutralization assay has been developed for evaluating neutralizing antibodies against SARS-CoV-2 in biosafety level 2 facilities. The key parameters for this assay were optimized, including cell types, cell numbers, virus inoculum. When tested against the SARS-CoV-2 pseudovirus, SARS-CoV-2 convalescent patient sera showed high neutralizing potency, which underscore its potential as therapeutics. The limit of detection for this assay was determined as 22.1 and 43.2 for human and mouse serum samples respectively using a panel of 120 negative samples. The cutoff values were set as 30 and 50 for human and mouse serum samples, respectively. This assay showed relatively low coefficient of variations with 15.9% and 16.2% for the intra- and inter-assay analyses respectively. Taken together, we established a robust pseudovirus-based neutralization assay for SARS-CoV-2 and are glad to share pseudoviruses and related protocols with the developers of vaccines or therapeutics to fight against this lethal virus.

## Introduction

Coronavirus is a member of coronavirus subfamily Coronaviridae. Its host is rich and varied. In addition to human beings, coronavirus can also infect many kinds of mammals and birds. Some coronaviruses can cause diseases in humans, livestock and poultry. Before 2019, there are six known human coronaviruses, belonging to α-coronavirus and β-coronavirus, causing respiratory diseases of different severity, including four common coronaviruses and two highly pathogenic emerging coronaviruses: severe acute respiratory syndrome coronavirus (SARS-CoV) and Middle East respiratory syndrome coronavirus (MERS-CoV). In December 2019, unexplained pneumonia (later named as coronavirus disease 2019, COVID-19) broke out in Wuhan, China [1–4]. The initial patient was related to a seafood wholesale market in Wuhan. A new type of β-coronavirus was found by unbiased high-throughput sequencing of samples from patients with pneumonia. A new type of coronavirus was isolated from human respiratory epithelial cells, which belongs to the subgenus Sabevirus of the subfamily Coronavirus [4]. Different from the previously isolated MERS-CoV and SARS-CoV, this virus is the seventh coronavirus that can infect humans and is named as SARS-CoV-2.

Coronavirus is an envelope virus with four structural proteins: spike (S) protein, membrane (M) protein, envelope (E) protein and nucleocapsid (N) protein [5–7]. S protein is responsible for the virus attachment and entry to the target cells, which initiate the infection process. S protein plays key roles in induction of protective humoral and cellular immunity during SARS-CoV infection. Consequently, the S protein was considered as the most attractive target for SARS-CoV vaccine and therapeutic development [8–11]. In face of the novel coronavirus pneumonia epidemic, a variety of approaches have been employed to develop prophylactic and therapeutic measures, including whole inactivated vaccine, subunit vaccine, RNA-based vaccine, viral vectored vaccines, monoclonal neutralizing antibodies, fusion inhibitors, most of which was designed to target the S protein. However, due to its high infectivity and pathogenicity, SARS-CoV-2 should be handled in biosafety level 3 (BSL-3) facilities, which has limited the development of anti-viral measures. The accessibility of the live virus stain is another major barrier to develop candidate vaccines and therapeutics.

To avoid dealing with infectious virus, we have developed a series of pseudovirus-based neutralization assays (PBNAs) for emerging and re-emerging viruses, including MERS-CoV [12], rabies virus [13], Ebola virus [14], Marburg virus [15], Lassa virus [16], Chikungunya virus [17], Nipah virus [18], Rift valley virus [19], and others. In this communication, we developed a PBNA for evaluation of anti-viral measures for SARS-CoV-2, which would be used to evaluate the inhibition of viral attachment and entry mediated by the S protein. The pseudoviruses and protocols for this PBNA are ready to be shared with research teams and manufactures engaging in the development of anti-viral products against SARS-CoV-2.

## Materials and methods

### Cells and samples

HepG2 (American Type Culture Collection [ATCC], HB-8065), Huh-7 (Japanese Collection of Research Bioresources [JCRB], 0403), 293T (ATCC, CRL-3216), Vero (ATCC, CCL-81), CHO (ATCC, CCL-61), and MDCK (ATCC, CCL-34) cells were maintained in high glucose DMEM (GIBCO) supplemented with 10% FBS (GIBCO), penicillin (100 IU/ml), streptomycin (100 μg/ml), and HEPES (20 mM) in a 5% CO_2_ environment at 37°C and passaged every 2–3 days.

VSV G specific antibody was generated by immunized Balb/c mouse with VSV G expressing plasmid at amount of 20 μg/mouse. Ten mice were immunized three times with two weeks interval. Two weeks after the final injection, serum samples from the ten mice were collected and pooled together as the VSV G specific antibody sample. The mice were housed and maintained in accordance with the relevant guidelines and regulations.

Two SARS-CoV-2 serum samples from convalescent patients, 2019-CS-1, 2019-CS-2 were generously provided by Mr. Qiang Gao from Sinovac Biotech Co., LTD. Written informed consents were obtained from all the volunteers.

### Production and titration of pseudoviruses

For pseudoviruses construction, spike genes from strain Wuhan-Hu-1 (GenBank: MN908947) were codon-optimized for human cells and cloned into eukaryotic expression plasmid pcDNA3.1 to generate the envelope recombinant plasmids pcDNA3.1.S2.

The pseudoviruses were produced and titrated using methods similar to Rift valley fever pseudovirus, as described previously [19,20]. For this VSV pseudovirus system, the backbone was provided by VSV G pseudotyped virus (G*ΔG-VSV) that packages expression cassettes for firefly luciferase instead of VSV-G in the VSV genome. Briefly, 293T cells were transfected with pcDNA3.1.S2 (30 μg for a T75 flask) using Lipofectamine 3000 (Invitrogen, L3000015) following the manufacturer’s instruction. Twenty-four hours later, the transfected cells were infected with G*ΔG-VSV with a multiplicity of four. Two hours after infection, cells were washed with PBS three times, and then new complete culture medium was added. Twenty-four hours post infection, SARS-CoV-2 pseudoviruses containing culture supernatants were harvested, filtered (0.45-μm pore size, Millipore, SLHP033RB) and stored at −70°C in 2-ml aliquots until use. The 50% tissue culture infectious dose (TCID_50_) of SARS-CoV-2 pseudovirus was determined using a single-use aliquot from the pseudovirus bank; all stocks were used only once to avoid inconsistencies that could have resulted from repeated freezing-thawing cycles. For titration of the SARS-CoV-2 pseudovirus, a 2-fold initial dilution was made in hexaplicate wells of 96-well culture plates followed by serial 3-fold dilutions (nine dilutions in total). The last column served as the cell control without the addition of pseudovirus. Then, the 96-well plates were seeded with trypsin-treated mammalian cells adjusted to a pre-defined concentration. After 24 h incubation in a 5% CO_2_ environment at 37°C, the culture supernatant was aspirated gently to leave 100 μl in each well; then, 100 μl of luciferase substrate (Perkinelmer, 6066769) was added to each well. Two min after incubation at room temperature, 150 μl of lysate was transferred to white solid 96-well plates for the detection of luminescence using a microplate luminometer (PerkinElmer, Ensight). The positive well was determined as ten-fold relative luminescence unit (RLU) values higher than the cell background. The 50% tissue culture infectious dose (TCID_50_) was calculated using the Reed–Muench method, as described previously [13,18,19].

### Western blot analysis

Seven millilitres of SARS-CoV-2 pseudoviruses with a titre of 1.86 × 10^5^ TCID_50_/ml were pelleted through a 25% sucrose cushion by ultra-centrifugation at 100,000× *g* for 3 h. The layers of supernatant and sucrose were removed, and the resulting viral pellets were re-suspended in 100 μl PBS. Sixty microlitre prepared pseudoviruses were mixed with 15 μl 6× SDS-sample buffer. The mixture was heated for 5 min at 100°C. Fifteen microlitre samples were subjected to SDS-PAGE and immunoblotting. The VSV pseudotyped virus was prepared with the same procedure and used as the pseudovirus negative control, cell culture medium as negative control. The incorporation of the spike protein on the pseudovirus surface was confirmed using Western bolt with SARS-CoV-2 convalescent serum sample as the detection antibody with a 500-fold dilution. Goat anti-human IgG (Jackson ImmunoResearch, 109-035-0030) was used with a 1:8000 dilution as the secondary antibody.

### Pseudovirus based neutralization assay

Neutralization was measured by the reduction in *luc* gene expression, as described previously for the HIV pseudovirus neutralization assay [21]. The 50% inhibitory dilution (EC_50_) was defined as the serum dilution at which the relative light units (RLUs) were reduced by 50% compared with the virus control wells (virus + cells) after subtraction of the background RLUs in the control groups with cells only. In brief, pseudovirus was incubated with serial dilutions of the test samples (six dilutions in a 3-fold step-wise manner) in duplicate for 1 h at 37°C, together with the virus control and cell control wells in hexaplicate. Then, freshly trypsinized cells were added to each well. Following 24 h of incubation in a 5% CO_2_ environment at 37°C, the luminescence was measured as described in the section for pseudovirus titration. The EC_50_ values were calculated with non-linear regression, i.e. log (inhibitor) vs. response (four parameters), using GraphPad Prism 8 (GraphPad Software, Inc., San Diego, CA, USA).

## Results

**Figure 1.**
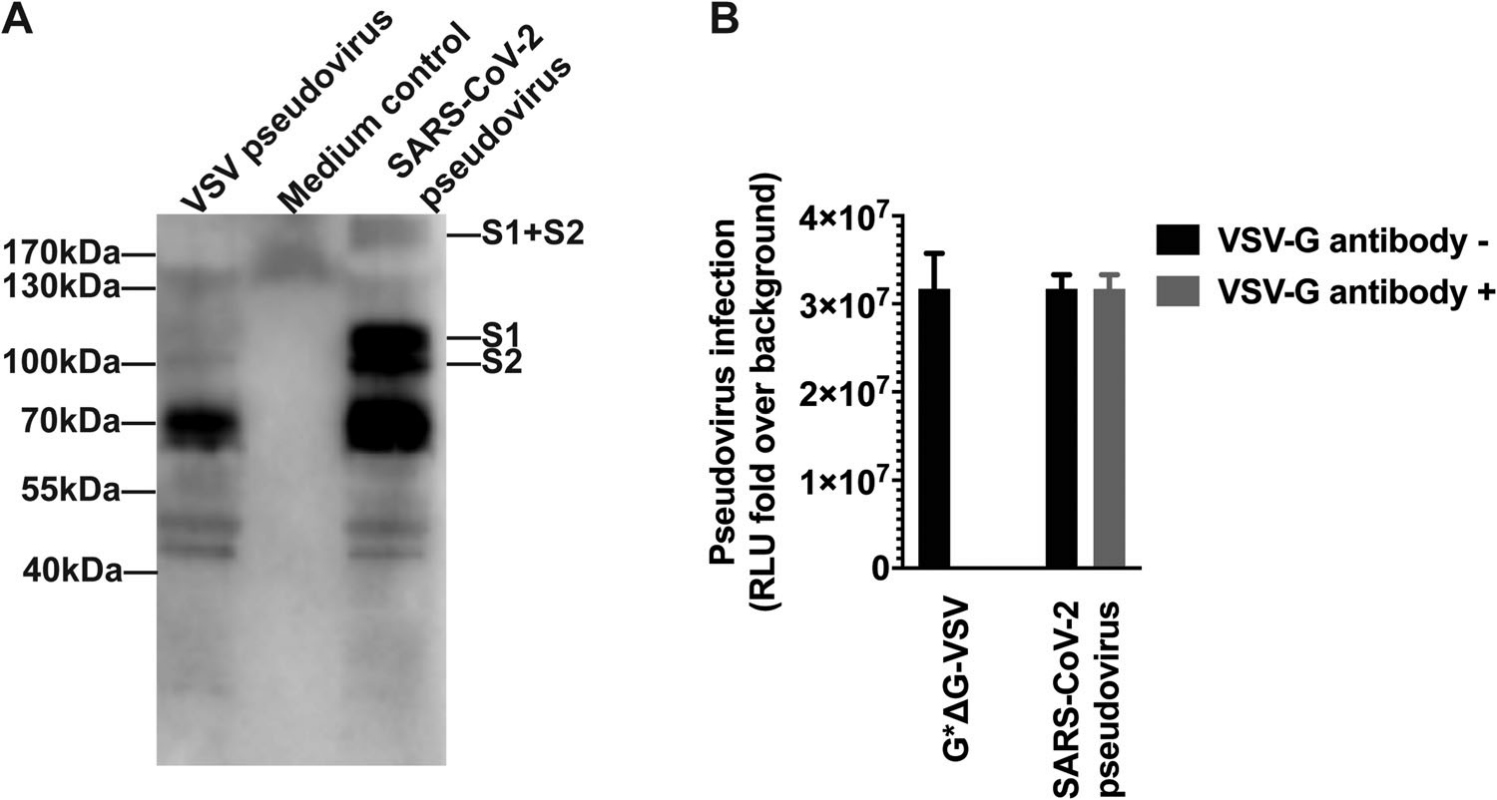
Verification of the incorporaiton of SARS-CoV-2 spike protein in the pseudovirus. The surface proteins of the particle were investigated using western blotting (A), from left to right lane showing VSV pseudovirus, medium control and SARS-CoV-2 pseudovirus. The VSV pseudotyped virus and cell culture medium were prepared with the same procedure and used as negative control and pseudovirus negative control. The SARS-CoV-2 pseudovirus, together with G*ΔG-VSV, were tested against VSV G specific antibody (B).

*Construction of the recombinant plasmid expressing SARS-CoV-2 spike protein*. To generate the SARS-CoV-2 S pseudotyped virus, we optimized the full-length S gene from strain Wuhan-Hu-1 (GenBank: MN908947) and inserted into the pcDNA3.1 to get pcDNA3.1.S2. The vesicular stomatitis virus (VSV) pseudovirus system was employed to produce the SARS-CoV-2 pseudovirus, which was expected to present the SARS-CoV-2 spike protein in the surface of the VSV particle [20]. To verify the incorporation of the spike protein, the surface protein in the SARS-CoV-2 pseudovirus was detected by using Western-blot with SARS-CoV-2 convalescent patient sera. As shown in Figure 1(A), specific bands could be found in the lanes of SARS-CoV-2 pseudovirus whilst no specific band was found in the medium control and VSV pseudovirus in the corresponding position, which was generated with the same procedure as SARS-CoV-2 pseudovirus. Monomer S protein (S1 + S2) were observed at position of about 190 kDa. The results confirmed that the 110 kDa and 80 kDa polypeptides correspond to the S1 and S2 domains, respectively. Bands at position of around 70 kDa were observed in both lanes for VSV and SARS-CoV-2 pseudovirus, which may be attributed to the shared VSV core for these two pseudoviruses.

**Figure 2.**
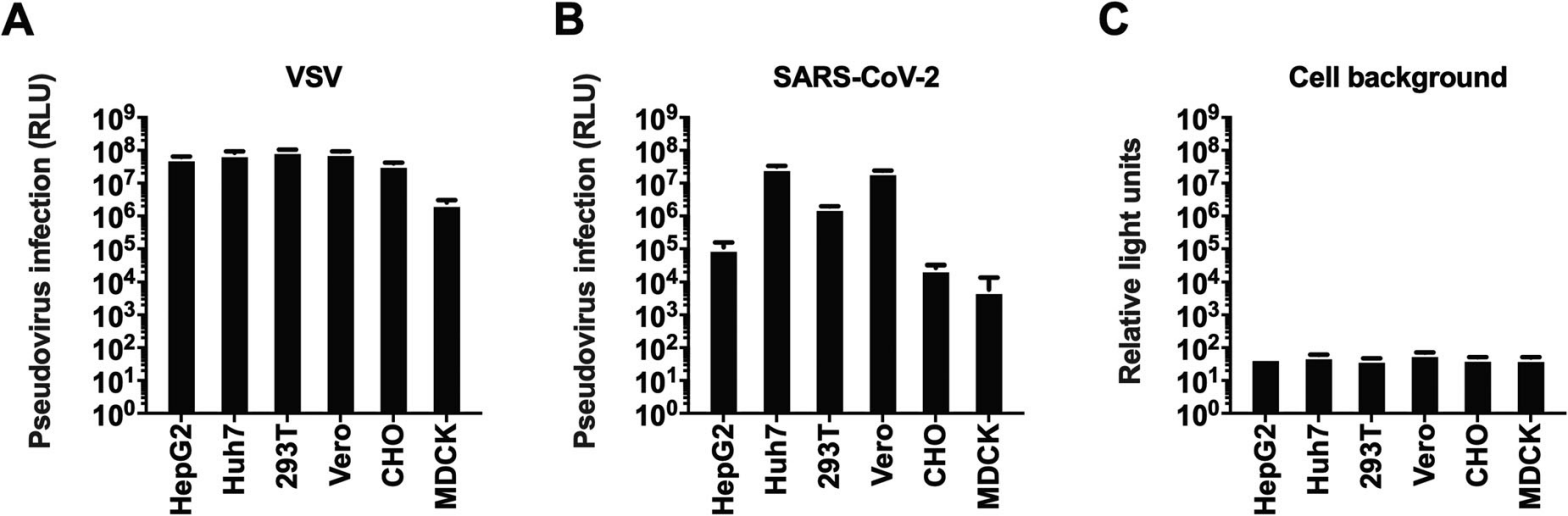
Selection of the cell line. Six types of cells were tested for VSV (A), and SARS-CoV-2 (B) pseudoviruses. The y-axis showed the absolute RLU value detected 24 h after pseudovirus infection. Cell backgrounds without pseudovirus infection were shown in figure C.

The SARS-CoV-2 pseudovirus, together with G*ΔG-VSV, were tested against VSV G specific antibody (Figure 2(B)). The SARS-CoV-2 pseudovirus could not been neutralized by the VSV G serum at a dilution of 1:100, with almost complete inhibition for G*ΔG-VSV infection. It is indicated that almost no G*ΔG-VSV was mixed in the SARS-CoV-2 pseudovirus stock.

### Optimization of SARS-CoV-2 PBNA

Having generated the SARS-CoV-2 S pseudotyped virus, we next set out to investigate the cell types for pseudovirus entry. The wide-type SARS-CoV-2 has been successfully isolated in both Vero (monkey kidney cell) and Huh7 (human hepatoma cell) [22]. Two similar cell lines, 293T (human kidney cell, similar to Vero) and Hep G2 (human hepatoma cell, similar to Huh7) were also included for the target cell investigation. In addition, another two mammal cell lines were also employed for target cell selection, including CHO (Chinese hamster ovary cell) and MDCK (dog kidney cell), which were widely employed in recombinant protein [23] or vaccine [24] production. Taken together, six types of cells were tested (Figure 2). As expected, all the cell lines showed high susceptibility to VSV pseudotyped virus. Huh7, 293T and Vero showed high susceptibility for SARS-CoV-2 pseudotypes, which was similar to SARS-CoV reported previously [25]. Huh7 cells were identified as the best cell substrate for SARS-CoV-2 pseudovirus infection, yielding the highest signals.

**Figure 3.**
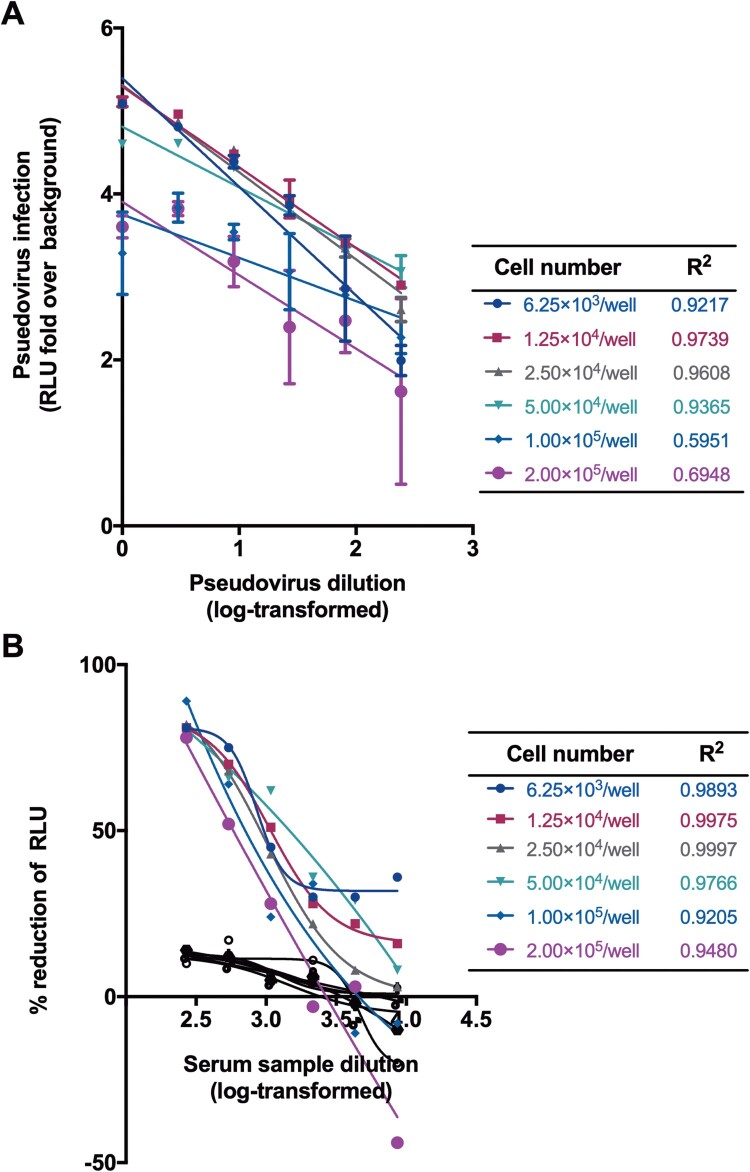
Optimization of cell numbers for neutralization. (A) Effect of cells numbers on the titration of pseudovirus. The y-axis showed the RLU fold over the cell background. (B) Effect of cells numbers on the neutralization. Cell numbers and corresponding *R*^2^ values were list in the tables. A serum sample from a healthy individual was tested as negative control shown in black lines and symbols.

We next determined the optimal number of Huh7 cells for SARS-CoV-2 pseudovirus infection. Towards this end, we titrated the SARS-CoV-2 pseudotyped virus at a range of cell numbers (6.25 × 10^3^–2.00 × 10^5^/well). The highest titre was observed in a range of 1.25 × 10^4^–2.50 × 10^4^/well when equal amount of pseudovirus preparations was tested (Figure 3(A)). It is noted that the linear correlation coefficients (*R*^2^) obtained at cell inoculum from 1.25 × 10^4^ to 2.50 × 10^4^/well were found to be greater than 0.96, revealing an excellent linear curve fitting. Moreover, when neutralization assays were performed with different cell inocula, the EC_50_ values were similar with cell numbers ranging from 6.25 × 10^3^ to 5.00 × 10^4^/well (Figure 3(B)). However, when the input cells numbers were more than 5.00 × 10^4^/well, the *R*^2^ values for both titration and inhibition assay would decrease dramatically. Given these findings, we chose 5.00 × 10^4^/well for subsequent experiments. Although non-specific responses were observed in varied cell number conditions, the inhibition of pseudovirus have not reached 50% for normal human serum samples (Figure 3(B)).

**Figure 4.**
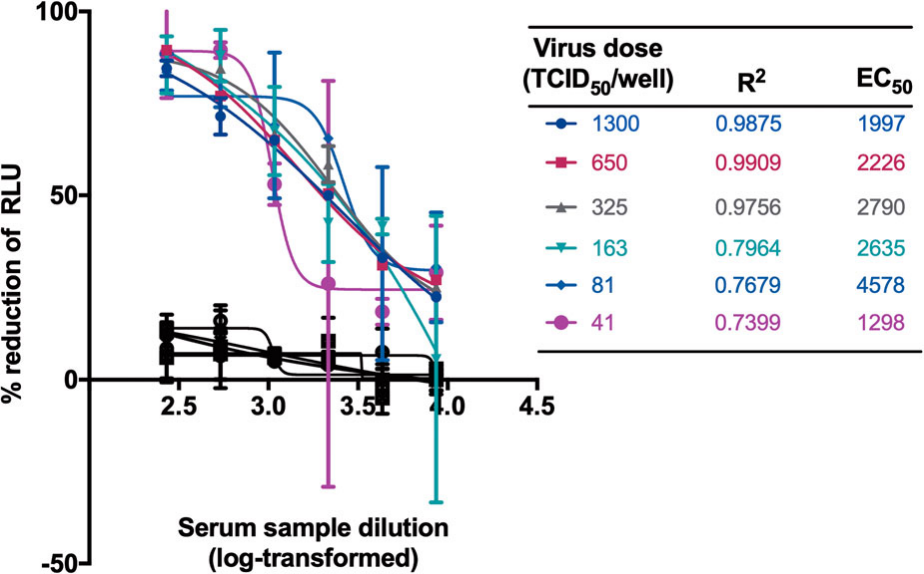
Optimization of virus dose for neutralization. The viral inocula with a dose range starting from 41 to 1300 TCID_50_/well were tested for SARS-CoV-2 PBNA. A serum sample from a healthy individual was tested as negative control shown in black lines and symbols.

We next tested the viral inocula with a dose range starting from 41 to 1300 TCID_50_/well for SARS-CoV-2 PBNA. As expected, the absolute EC_50_ values decreased gradually with the increasing amounts of the viral inocula (Figure 4). When the virus dose was lower than 325 TCID_50_/well, the curve fit value would decrease significantly. Therefore, the viral inocula of 650 TCID_50_/well was used as the optimal dose. Although non-specific responses were observed in varied viral inocula, the inhibition rate of pseudovirus have not reached 50% for normal human serum samples (Figure 4).

### Establishment and validation of the SARS-CoV-2 PBNA

**Figure 5.**
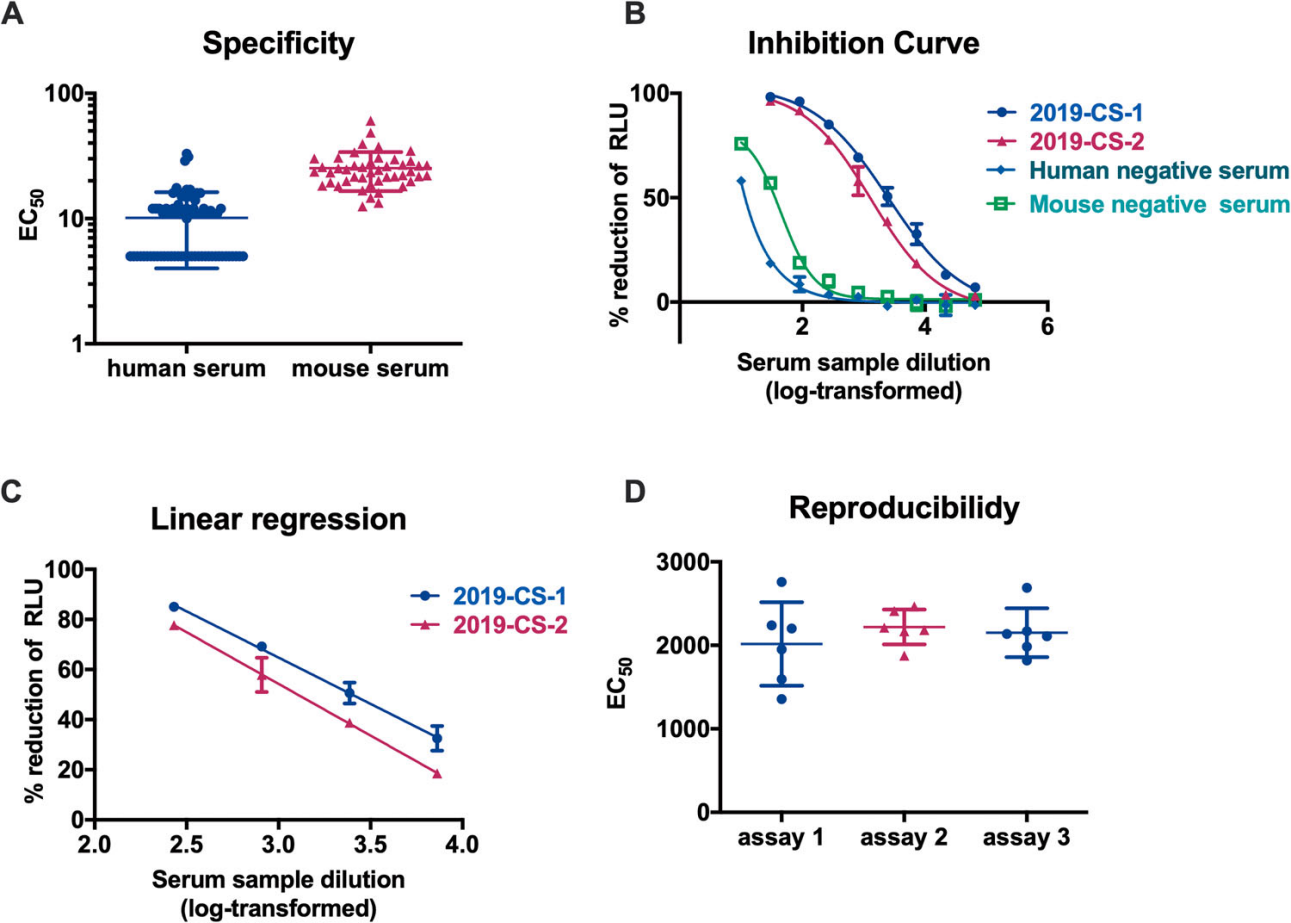
Validation of the SARS-CoV-2 PBNA. (A) Specificity of the PBNA. A negative sample panel of seventy-four human and forty-six mouse serum samples were used to determine the specificity of this assay. (B) Inhibition curves for two SARS-CoV-2 convalescent patient serum samples and two negative serum samples derived from human and mouse. Typical four-parameter inhibition curves were observed for these two samples between log-transformed dilution and inhibition rate. The initial dilutions for positive and negative samples were 1:30 and 1:10 respectively, followed by 3-fold serial dilution. (C) Linear range for the PBNA. When the inhibition rate is in the range of 20%–80%, there is a linear relationship between the readout of the test sample and its dilution. (D) Reproducibility of the PBNA. A mixture of two SARS-CoV-2 coalescent patient serum samples was tested a total of 18 times on individual plates in three independent runs.

As the SARS-CoV-2 PBNA assay would be intended to be used for the analyses of both human and animal samples, a negative sample panel of seventy-four human and forty-six mouse sera were used to determine the specificity of this assay, with an initial dilution of 1:10 followed by 3-fold serial dilution. We first established the limit of detection (LOD) for SARS-CoV-2 PBNA assay. As shown in Figure 5(A), when assayed by PBNA, human negative serum samples showed relatively low background compared to the mouse negative samples. When the mean titre value of the negative samples +1.96 standard deviation (SD) was used to calculate the limit of detection (LOD), the LOD was 22.1 for human serum samples and 42.3 for mouse serum samples. The cutoff values were set as 30 and 50 for human and mouse serum samples, respectively. The non-specific background of mouse sera was higher than human serum samples, which was unexpected. The reason of the higher background for mouse sera would be investigated in future studies.

Linear range means that there is a direct correlation between the signal and the concentration of the substance within a specific range. To determine the linear range of the SARS-CoV-2 PBNA, two SARS-CoV-2 convalescent patient serum samples were tested against the pseudovirus. Typical four-parameter inhibition curves were observed for these two positive samples between log-transformed dilution and inhibition rate (Figure 5(B)). When the inhibition rate is in the range of 20%–80%, there is a linear relationship between the readout of the test sample and its dilution for the positive samples, *R*^2 ^> 0.99 (Figure 5(C)). Non-specific inhibition was observed for both human and mouse negative serum samples in a lower dilution (Figure 2(B)).

In order to test the reproducibility of the PBNA, two SARS-CoV-2 coalescent patient serum samples were pooled together and used to test against the SARS-CoV-2 pseudovirus. This sample was tested a total of 18 times on individual plates in three independent runs (Figure 5(D)). The average coefficient of variation (CV) for the intra- and inter-assay was 15.9% and 16.2% respectively, which was acceptable for a cell-based assay.

Collectively, these data indicate that a robust PBNA assay has been established to test entry inhibition for SARS-CoV-2, which could be used for evaluation of candidate vaccine and therapeutics targeting the virus entry.

## Discussion

Currently, the evaluation assays for the SARS-CoV-2 vaccines and therapeutics require the use of isolated live virus which must be handled at level 3 biocontainment facility. In addition, the live virus assay takes at least three days and is known to be labour intensive. Clearly, exploring alternative methods should facilitate the development and evaluation of SARS-CoV-2 vaccines and therapeutics targeting virus entry of the target cells.

Pseudovirus-based neutralizing assays (PBNA) offer great advantages over the wide-type virus-based methods because they are versatile and much safer to handle. The versatility of pseudovirus is achieved by pseudotyping the virus with different outer membrane proteins or envelope proteins, mimicking the infectious process of the live virus providing the envelope proteins [26,27]. Pseudovirus is much safer because the virus is essentially devoid of virulent viral components and involves in a single round of replication.

With respect to validation of neutralization assay, PBNA is a sensitive, accurate, reproducible and robust method. Compared with the live virus assay, PBNA is more objective and less labour intensive as the data were obtained through luminescent reading, while the operator of live virus assay has to read the results manually under the microscope. However, the EC_50_ values of serum samples would change with the variation of the pseudovirus inoculum. It is important to fix the pseudovirus dose to make the results comparable between different laboratories. Besides, antibody standard development should be scheduled in near future to minimize the variation of data obtained from different teams.

In short, the absence of lethal wide type virus in the experimental procedure could greatly facilitate the development of SARS-CoV-2 vaccine and therapeutics. We would like to provide SARS-CoV-2 pseudovirus with related protocols to vaccine and therapeutic developers for the evaluation of their candidate products.
